# National introduction of one-anastomosis gastric bypass in the UK National Bariatric Surgery Registry: a cohort study

**DOI:** 10.1097/JS9.0000000000002005

**Published:** 2024-09-23

**Authors:** Andrew C. Currie, Alan Askari, Chetan Parmar, James Byrne, Ahmed R. Ahmed, Chris M. Pring, Omar A. Khan, Peter K. Small, Kamal Mahawar

**Affiliations:** a Department of Upper Gastrointestinal and Bariatric Surgery, University Hospitals Derby and Burton, Derby, UK; b Department of Bariatric Surgery, Bedfordshire Hospitals NHS Trust, Luton, UK; c Department of Upper GI and Bariatric Surgery, Whittington Health NHS Trust, London, UK; d Department of Upper GI and Bariatric Surgery, University Hospital Southampton, Southampton, UK; e Department of Surgery and Cancer, Imperial College London, London, UK; f Department of Upper Gastrointestinal and Bariatric Surgery, University Hospitals Sussex (St Richard’s Hospital), Chichester, UK; g Department of Bariatric Surgery, St George’s University Hospitals NHS Foundation Trust, London, UK; h Department of General Surgery, Sunderland Royal Hospital, Sunderland, UK

**Keywords:** bariatric surgery, national registry, one-anastomosis gastric bypass (OAGB), postoperative morbidity, weight loss

## Abstract

**Aim:**

There is a paucity of evidence regarding the national introduction of newer bariatric metabolic surgery procedures. This study assessed the impact of introducing one-anastomosis gastric bypass (OAGB) in bariatric surgical practice in the UK on 30-day postoperative morbidity and early postoperative weight loss.

**Methods:**

Patients who underwent primary BMS in the UK National Bariatric Surgical Registry (2010–2019) were identified. Patient characteristics, 30-day postoperative morbidity, and 12-month total body weight loss (TBWL) were also assessed. Multivariate regression was performed for associations between 30-day postoperative morbidity and 12-month TBWL, with SG as a reference. Learning effects were assessed by factoring in the institutional OAGB caseload (0–24/25–49/50+ cases).

**Results:**

A total of 59 226 patients underwent primary BMS during the study period (RYGB, 38 434; SG, 24 702; AGB, 12 627; OAGB, 3408; and others, 276). The 30-day postoperative morbidity was lower for OAGB 1.8% (51/2802) compared to RYGB 4.2% (1391/32 853) and SG 3.4% (725/21 333) but higher than AGB 1.2% (123/9915), while on multivariate regression, OAGB was associated with reduced morbidity once the institution caseload exceeded 50 operations (OR 0.35 (95% CI: 0.22–0.56; *P*<0.001) and no statistical difference to SG at lesser caseloads. Overall, 12-month greater than 25% TBWL was seen in 69.4% (27 736/39 971) (RYGB: 82.9% (17 617/21 246)), SG: 65.4% (7383/11 283)), AGB: 23.9% (1382/5572)) and OAGB: 82.9% (1328/1601)). On multivariate regression, OAGB was associated with the highest 12-month TBWL once the institution caseload exceeded 50 operations (OR 3.47 (95% CI 2.75–4.39; *P*<0.001).

**Conclusion:**

OAGB has been safely implemented in UK national bariatric surgery practice. It has lower reported postoperative morbidity and comparable weight loss to RYGB or SG, despite being offered to patients with more severe and complex obesity.

Highlights

- One-anastomosis gastric bypass (OAGB) exhibited lower postoperative morbidity rates than traditional procedures like RYGB and SG, implying an enhanced safety profile.
- Despite treating more severe obesity cases, OAGB demonstrated weight loss outcomes comparable to RYGB and SG, achieving the highest 12-month total body weight loss in institutions performing over 50 operations.
- The successful integration of OAGB into the UK’s national bariatric surgery practice highlights its safe implementation, offering improved safety and substantial weight loss outcomes even for complex obesity cases.

Bariatric metabolic surgery (BMS) improves mortality and morbidity outcomes in patients with obesity^1^ and has been shown to be cost-effective^2–4^. Additionally, BMS improves functional impairment and cardiovascular disease, and reduces cancer risk and mortality related to type 2 diabetes (T2DM)^5–8^. However, studies have demonstrated that only a small proportion of the eligible population receives surgical intervention for obesity-related metabolic diseases^9,10^. Therefore, while BMS is part of a well-established treatment intensification strategy for obesity-related comorbidities^11,12^; the role of individual operative approaches is less certain.

Data from International Federation for the Surgery of Obesity (IFSO) Registry recently reported that of the 413 048 primary BMS operations, sleeve gastrectomy (SG) was the most common followed by Roux-en-y gastric bypass (RYGB) and then one-anastomosis gastric bypass (OAGB)^13^. A previous network meta-analysis using both direct randomized trials and indirect evidence demonstrated that OAGB had comparable or higher postoperative weight loss than either RYGB or SG^14^. Guidance from both IFSO^15,16^, and recently ASMBS^17^, has approved RYGB, SG, and OAGB as suitable primary bariatric-metabolic operations. OAGB is the most novel intervention, first reported by Rutledge in 1997^18^. Literature published from both the IFSO Registry and the Israeli National Registry indicates increased use of OAGB in the surgical treatment of obesity^13,19^. The safe introduction of new surgical procedures into unrestricted clinical practice can be confounded by the learning effects for both operative techniques and perioperative care practices^20,21^. No study has yet considered the introduction of OAGB at the population or national registry levels.

The current study evaluated the introduction of OAGB in clinical practice in the UK National Bariatric Surgery Registry. The primary outcome was the impact of OAGB use on early postoperative morbidity, with secondary outcomes of the effect of OAGB on bariatric surgical practice in submitting centers and the comparison of OAGB and other procedures on early weight loss and comorbidity changes.

## Methods

### Study design

The study adhered to the guidelines outlined by the Strengthening the reporting of cohort, cross-sectional, and case–control studies in surgery (STROCCS, Supplemental Digital Content 1, http://links.lww.com/JS9/D475) guidelines^22^. The study has been retrospectively registered on ClinicalTrials.gov with the unique identifier NCT06167005 and can be accessed here: https://clinicaltrials.gov/study/NCT06167005.

### Study setting

The data utilized in this study were obtained from the National Bariatric Surgery Registry (NBSR), a comprehensive nationwide registry encompassing all BMS procedures conducted in the United Kingdom and the Republic of Ireland^23,24^. The registry included detailed preoperative, operative, and follow-up data.

### Study population

A total of 59 226 individuals who underwent primary bariatric surgery in the United Kingdom and Ireland over an 11-year period (January 2009 to December 2019) were included in this study. Exclusions were made for Individuals under 18 years of age, those undergoing revisional surgery (defined as conversion to another bariatric procedure), or gastric balloon insertion as the sole treatment were excluded.

### Data collection

The study collected data pertaining to preoperative demographic characteristics, anthropometric measurements, presence of comorbidities [including type 2 diabetes mellitus (T2DM), liver disease, cardiovascular disease, gastroesophageal reflux disease (GERD), asthma, hypertension, dyslipidemia, arthritis, and depression], ethnicity, surgery date, functional impairment (degree of impairment in physical functioning) and the specific bariatric metabolic procedure undertaken. Within the dataset, T2DM was classified based on disease stage options, such as no indication of T2DM, impaired glycemia or glucose tolerance, oral glucose-lowering medications, insulin treatment, or other injectable therapy. T2DM and hypertension diagnoses were assigned to patients who required one or more medications for each condition. GERD was recorded in individuals who reported reflux symptoms or received intermittent or daily medication for the disease. Baseline disease staging was evaluated using the American Society of Anesthesiologists (ASA) grade, Obesity Surgery-Mortality Risk Score (OS-MRS), and Edmonton Obesity Staging System (EOSS). The OS-MRS incorporates age, sex, BMI, hypertension, and known venous thromboembolism (VTE) risk factors to predict perioperative mortality^25^. EOSS, previously calculated using the NBSR dataset, predicts long-term mortality based on the severity of baseline obesity-related diseases^24,26^ (see Supplementary Table 1, Supplemental Digital Content 2, http://links.lww.com/JS9/D476).

### Procedure types

The study considered four types of bariatric-metabolic procedures: Roux-en-Y gastric bypass (RYGB), sleeve gastrectomy (SG), gastric banding (GB), and one-anastomosis gastric bypass (OAGB). The operative type was coded prospectively by the operating surgeon. Until 2016, when NBSR introduced a specific entry for OAGB, OAGB cases were identified from contemporaneous free text reporting by the operating surgeon (Appendix 1, Supplemental Digital Content 3, http://links.lww.com/JS9/D477).

### Outcomes

The primary outcomes of the study were the development of any complications within 30 days of the operation, including severe complications, which were defined as death, unplanned ITU admission, or reoperation within 30 days of the primary operation. Only patients with a recorded complication before 30 days or any recorded entry at or after 30 days were included in this analysis. Secondary outcomes included percentage excess weight loss at 12 months, impact of OAGB on practice within submitting institutions, and comorbidity resolution at 12 months. Postoperative weight loss is presented as a percentage of total weight loss (%TWL), which was defined as (preoperative weight – postoperative weight)/preoperative weight ×100^27^. Institutions were defined as ‘OAGB performers’ if they had performed 10 OAGB procedures during the study period. This was done to gain a view on planned changes in hospital practice and to avoid coding unplanned or reactive OAGB formation due to intraoperative circumstances. Both weight loss and comorbidity resolution were calculated at 12 months, with endpoint data accepted between 6 and 24 months, where coded.

### Statistical analysis

Patient demographic and clinical characteristics were compared across groups using the Pearson *χ* ^2^ test for categorical nominal data and the Mann–Whitney *U*-test for continuous data. Univariate and multivariate logistic regression models were employed to identify factors associated with 30-day any and major complications, as well as 50% excess weight loss and comorbidity resolution at 12 months, and the results were reported as odds ratios (OR) with corresponding 95% CI. Additionally, baseline factors and outcomes were compared across different procedure types using analysis of variance or *χ* ^2^ tests, as appropriate. Further adjusted comparisons of factors associated with procedure selection were conducted using multinomial logistic regression, with sleeve gastrectomy serving as the reference category because of its widespread adoption as the most common bariatric metabolic surgery globally. All statistical analyses were performed using SPSS version 29 software (IBM Corp.). Statistical significance was set at *P*<0.05.

## Results

### Procedures over time

**Figure 1:**
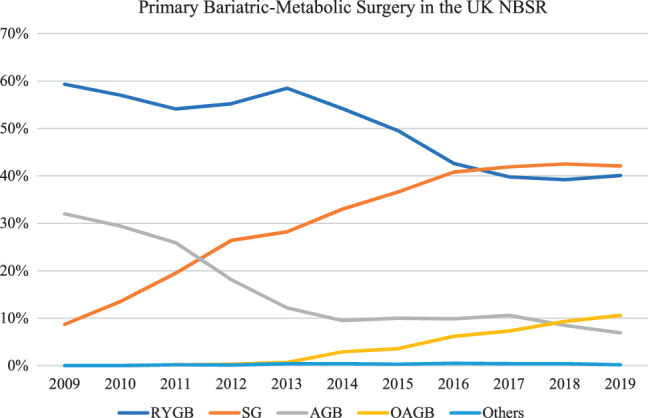
Trends in procedure type for patients undergoing primary bariatric-metabolic surgery within the NBSR 2009–2019.

A total of 59 226 patients underwent primary bariatric metabolic surgery during the study period. Thirty-eight thousand four hundred thirty-four patients received RYGB, 24 702 underwent SG, 12 627 underwent AGB, 3408 underwent OAGB, and 276 underwent other procedures. The procedure distribution changed over time, with considerable reductions in AGB and, to a much lesser extent, in RYGB (Fig. 1). The use of SG has considerably increased. OAGB was first reported in 2012, and by 2019, represented just over 10% of primary operations in the UK NBSR.

### Differences in patient demographics between different procedures

The distribution of patient characteristics according to procedure type is shown in Table 1. There are considerable differences between the populations undergoing RYGB, SG, or OAGB and those undergoing AGB. The OAGB population has a higher proportion of older adults (aged >60 years), those with liver disease, and those with higher stages of both the OS-MRS score and ASA.

**Table 1:** Characteristics of patients undergoing Primary BMS within the NBSR (*N*=59 226).

	RYGB (*n*=38 434)	SG (*n*=24 702)	AGB (*n*=12 617)	OAGB (*n*=3408)	Others (*n*=276)	*P*
Mean age (years) (±SD)	45.2 (10.9)	45.0 (11.6)	43.6 (11.5)	45.7 (11.3)	45.5 (9.8)	<0.001
Age distribution (years)						
18–30	4047 (10.5%)	3011 (12.2%)	1761 (14.0%)	362 (10.6%)	21 (7.6%)	<0.001
31–40	8610 (22.4%)	5619 (22.7%)	3140 (24.9%)	782 (22.9%)	67 (24.3%)	<0.001
41–50	13 038 (33.9%)	7657 (31.0%)	4081 (32.3%)	1034 (30.3%)	108 (39.1%)	<0.001
51–60	9938 (25.9%)	6300 (25.5%)	2681 (21.2%)	925 (27.1%)	63 (22.8%)	<0.001
60+	2801 (7.3%)	2115 (8.6%)	954 (7.6%)	305 (8.9%)	17 (6.2%)	<0.001
Sex (male)	8274 (21.5%)	6008 (24.3%)	1965 (15.6%)	806 (23.7%)	77 (27.9%)	<0.001
Caucasian ethnicity	31 237 (86.3%)	19 087 (81.3%)	9934 (85.7%)	2888 (86.1%)	237 (88.8%)	<0.001
Mean initial BMI (kg/m^2^) (±SD)	48.0 (7.6)	48.2 (9.0)	43.2 (7.8)	47.8 (8.1)	45.6 (11.6)	<0.001
Initial BMI distribution (kg/m^2^)						
<35	437 (1.2%)	653 (2.7%)	1035 (8.5%)	50 (1.5%)	57 (21.0%)	<0.001
35–39.9	3697 (9.9%)	3353 (14.0%)	2875 (23.5%)	387 (11.5%)	63 (23.2%)	<0.001
40–49.9	19 273 (51.6%)	11 077 (46.2%)	6033 (49.3%)	1747 (52.1%)	86 (31.6%)	<0.001
≥50	13 948 (37.3%)	8886 (37.1%)	2302 (18.8%)	1170 (34.9%)	66 (24.3%)	<0.001
Asthma	7430 (20.5%)	4496 (18.7%)	1617 (14.2%)	628 (18.7%)	30 (11.0%)	<0.001
Cardiovascular disease	1911 (5.3%)	1155 (4.8%)	337 (3.0%)	132 (3.9%)	11 (4.0%)	<0.001
Depression	10 070 (26.2%)	6382 (25.8%)	2420 (19.2%)	850 (24.9%)	72 (26.1%)	<0.001
GERD	9922 (28.3%)	4254 (18.3%)	1806 (16.5%)	615 (18.7%)	35 (13.1%)	<0.001
Hypertension	14 216 (38.6%)	8220 (34.1%)	3159 (26.4%)	1102 (32.8%)	76 (27.8%)	<0.001
Liver disease	2314 (6.6%)	1648 (7.0%)	407 (3.7%)	274 (8.3%)	15 (5.6%)	<0.001
Musculoskeletal pain	11 127 (31.0%)	6977 (29.4%)	2209 (19.7%)	1067 (32.1%)	57 (20.9%)	<0.001
OSA	8808 (24.0%)	5672 (23.6%)	1269 (10.7%)	761 (22.6%)	38 (13.9%)	<0.001
PCOS	3113 (11.3%)	1970 (11.1%)	742 (7.9%)	283 (11.3%)	12 (6.1%)	<0.001
T2DM	10 900 (29.6%)	4753 (19.8%)	1489 (12.5%)	862 (25.6%)	52 (19.1%)	<0.001
VTE risk factors	6229 (17.2%)	3957 (16.5%)	1687 (15.1%)	1009 (30.0%)	89 (32.6%)	<0.001
Functional status						
Wheelchair / housebound	866 (2.4%)	761 (3.2%)	127 (1.2%)	86 (2.6%)	10 (3.7%)	<0.001
Can manage ½ flight stairs	5875 (16.5%)	4564 (19.2%)	1405 (12.7%)	805 (24.5%)	60 (22.1%)	<0.001
Can manage 1 flight stairs	17 950 (50.5%)	10 693 (45.0%)	4843 (43.9%)	1445 (44.0%)	129 (47.4%)	<0.001
Can manage 3 flight stairs	10 873 (30.6%)	7764 (32.6%)	4649 (42.2%)	951 (28.9%)	73 (26.8%)	<0.001
ASA grade						
I	2718 (7.1%)	2169 (8.8%)	2613 (20.7%)	362 (10.6%)	76 (27.5%)	<0.001
II	21 690 (56.4%)	13 371 (54.1%)	7132 (56.5%)	1454 (42.7%)	133 (48.2%)	<0.001
III	11 349 (29.5%)	8172 (33.1%)	1461 (11.6%)	1491 (43.8%)	54 (19.6%)	<0.001
IV	174 (0.5%)	155 (0.6%)	27 (0.2%)	56 (1.6%)	9 (3.3%)	<0.001
OS-MRS score						
Low risk (0–1)	16 837 (51.3%)	11 786 (52.8%)	6286 (67.1%)	1581 (48.8%)	119 (46.1%)	<0.001
Moderate risk (2–3)	14 432 (44.0%)	9197 (41.2%)	2857 (30.5%)	1425 (44.0%)	125 (48.4%)	<0.001
High risk (4–5)	1521 (4.6%)	1360 (6.1%)	227 (2.4%)	233 (7.2%)	14 (5.4%)	<0.001
EOSS stage*						
Stage 0	2884 (8.8%)	2362 (10.6%)	2154 (21.8%)	288 (9.0%)	35 (13.7%)	<0.001
Stage 1	2342 (7.1%)	2122 (9.5%)	1274 (12.9%)	312 (9.7%)	39 (15.2%)	<0.001
Stage 2	25 397 (77.2%)	16 124 (72.4%)	6113 (61.9%)	2409 (75.1%)	163 (63.7%)	<0.001
Stage 3	1505 (4.6%)	977 (4.4%)	239 (2.4%)	119 (3.7%)	9 (3.5%)	<0.001
Stage 4	768 (2.3%)	698 (3.1%)	101 (1.0%)	80 (2.5%)	10 (3.9%)	<0.001

Column proportions were statistically compared using the *χ* ^2^ test, and column means (and SD) were compared using ANOVA.

Comorbidities were defined as % on daily medication or treatment.

ASA, American Society of Anesthesiologists Score; EOSS, edmonton obesity staging system; GORD, gastroesophageal reflux disease; OSA, obstructive sleep apnea; OS-MRS, Obesity Surgery-Mortality Risk Score; PCOS, polycystic ovary syndrome; T2DM, type 2 diabetes mellitus; VTE, venous thromboembolism.

### Technical elements in OAGB

**Figure 2:**
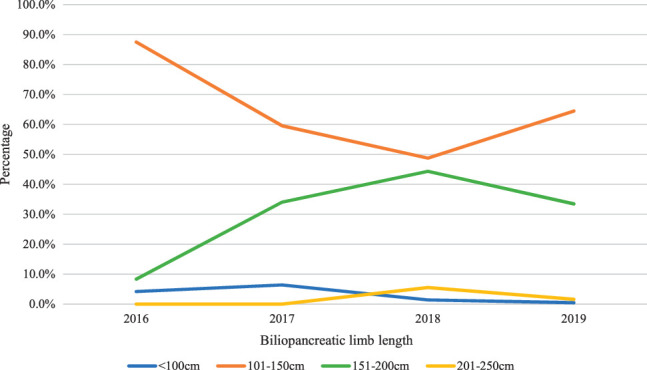
Change in biliopancreatic limb length for patients receiving OAGB within the NBSR*. *Data reported from 2016 onwards as fewer than 50 procedures annually prior to this study point

The specific technical elements of OAGB were recorded in the registry from 2016. The biliopancreatic (BP) limb length changed over the course of the study, with a reduced use of 200 cm length and greater use of 100–150 cm BP limb length (Fig. 2). Whether a bougie was used was reported in 1130 patients, with 121/1130(10.7%) reporting no bougie use and nearly 80% reporting 34 French or wider diameter bougie use. Linear stapled construction of one-anastomosis was nearly universal (1371/1380; 99.3%). Closure of the mesenteric (Peterson’s) space was reported in of 343/1067 (32.1%) cases.

### Rates of complications and associations with 30-day any and severe complications

**Figure 3:**
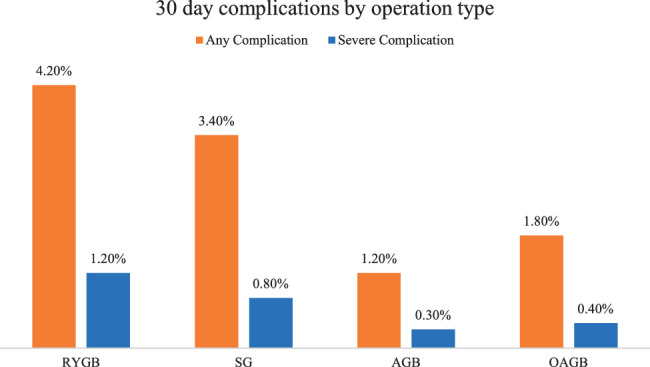
30-day complications type by OAGB, RYGB, SG, and AGB.

The overall rates for the four procedures are shown in Figure 3, with OAGB having lower overall rates of any and severe complications than RYGB and SG. The multivariate regressions of any complications and severe complications are presented in Tables 2 and 3, respectively. After adjustment, OAGB was associated with a reduced risk of severe complications, even during the learning phase. An increasing ASA grade is associated with an increased risk of complications and severe complications. Non-Caucasian ethnicity is associated with an increased risk of complications, but not severe complications. Increased age is associated with an increased risk of severe complications, but not of any complications. A more recent year of surgery was associated with a reduced risk of complications, but not severe complications. No technical element was associated with the risk of perioperative complications.

**Table 2:** Multivariate logistic regression of factors associated development of any complications up to 30 days postoperatively for patients undergoing primary BMS in the NBSR.

	Adjusted OR (95% CI)	*P*
Procedure		
Sleeve gastrectomy	Ref	
OAGB (Cases 0–24)	0.61 (0.32–1.16)	0.133
OAGB (Cases 25–49)	0.63 (0.32–1.24)	0.184
OAGB (Case 50+)	0.35 (0.22–0.56)	<0.001
RYGB	1.22 (1.11–1.34)	<0.001
AGB	0.51 (0.42–0.60)	<0.001
Other	1.83 (0.88-3.80)	0.103
Year of surgery		
2009–2012	Ref	
2013–2015	0.88 (0.80–0.97)	0.010
2016–2019	0.84 (0.76–0.94)	0.002
Funding type		
Self-pay	Ref	
NHS	1.31 (1.16–1.48)	<0.001
Age at surgery (years)		
18–30	(Ref)	
31–40	0.93 (0.80–1.09)	0.358
41–50	0.93 (0.80–1.08)	0.317
51–60	1.00 (0.85–1.17)	0.993
60+	1.04 (0.85–1.27)	0.690
Sex		
Male	0.95 (0.85–1.05)	0.275
Ethnicity		
Non-Caucasian	0.92 (0.82–1.04)	0.185
Initial BMI (kg/m^2^)		
<35	(Ref)	
35–39.9	1.10 (0.78–1.56)	0.574
40–49.9	1.13 (0.81–1.58)	0.460
≥50	1.22 (0.87–1.71)	0.245
Medical conditions		
Asthma	1.23 (1.12–1.35)	<0.001
Cardiovascular disease	1.30 (1.10–1.53)	0.002
Depression	1.00 (1.00–1.00)	0.010
Dyslipidaemia	1.04 (0.94–1.16)	0.442
GERD	1.21 (1.10–1.33)	<0.001
Hypertension	1.01 (0.92–1.11)	0.849
Liver disease	1.18 (1.02–1.37)	0.023
Musculoskeletal pain	1.19 (1.09–1.30)	<0.001
OSA	1.07 (0.97–1.18)	0.184
VTE risk factors	0.86 (0.77–0.95)	0.005
T2DM	1.09 (0.98–1.20)	0.101
ASA		
1	Ref	
2	1.06 (0.91–1.24)	0.451
3	1.20 (1.00–1.43)	0.047
4	2.33 (1.53–3.55)	<0.001
Impaired functional status		
Cannot climb three flights of stairs or more	0.97 (0.88–1.07)	0.533

Impaired functional status is defined as inability to climb three flights of stairs.

GERD, Gastroesophageal Reflux Disease; OR, odds ratio; OSA, obstructive sleep apnea; Ref, reference; VTE, venous thromboembolism.

**Table 3:** Multivariate logistic regression of factors associated development of severe complications (30 days death/unplanned ITU admission/30 days reoperation) for patients undergoing primary BMS in the NBSR.

	Adjusted OR (95% CI)	*P*
Procedure		
Sleeve gastrectomy	Ref	
OAGB (Cases 0–24)	0.54 (0.13–2.19)	0.388
OAGB (Cases 25–49)	0.30 (0.04–2.17)	0.234
OAGB (Case 50+)	0.10 (0.01–0.70)	0.020
RYGB	1.55 (1.30–1.86)	<0.001
AGB	0.65 (0.48–0.88)	0.005
Other	4.61 (1.62–13.10)	0.004
Year of Surgery		
2009–2012	Ref	
2013–2015	0.77 (0.65–0.92)	0.003
2016–2019	0.85 (0.70–1.03)	0.101
Funding type		
Self-pay	Ref	
NHS	1.00 (0.81–1.24)	0.985
Age at surgery (years)		
18–30	(Ref)	
31–40	1.27 (0.95–1.69)	0.101
41–50	1.06 (0.80–1.42)	0.667
51–60	1.42 (1.06–1.92)	0.020
60+	1.47 (1.02–2.12)	0.039
Sex		
Male	1.01 (0.84–1.21)	0.926
Ethnicity		
Non-Caucasian	0.92 (0.73–1.16)	0.488
Initial BMI (kg/m^2^)		
<35	(Ref)	
35–39.9	0.87 (0.51–1.48)	0.604
40–49.9	0.74 (0.44–1.24)	0.250
≥50	0.80 (0.47–1.35)	0.404
Medical conditions		
Asthma	1.06 (0.89–1.27)	0.495
Cardiovascular disease	1.05 (0.76–1.46)	0.766
Depression	0.99 (0.99–1.00)	<0.001
Dyslipidaemia	0.97 (0.80–1.18)	0.773
GERD	1.13 (0.96–1.34)	0.138
Hypertension	0.98 (0.82–1.17)	0.832
Liver disease	1.03 (0.77–1.37)	0.850
Musculoskeletal pain	1.09 (0.92–1.28)	0.324
OSA	0.97 (0.81–1.17)	0.770
VTE risk factors	0.86 (0.70–1.05)	0.133
T2DM	0.91 (0.75–1.10)	0.323
ASA		
1	Ref	
2	1.18 (0.89–1.55)	0.247
3	1.27 (0.93–1.74)	0.131
4	2.59 (1.18–5.68)	0.017
Impaired functional status		
Cannot climb three flight of stairs or more	0.79 (0.67–0.93)	0.004

Impaired functional status defined as inability to climb three flights of stairs.

GERD, Gastroesophageal Reflux Disease; OR, odds ratio; OSA, obstructive sleep apnea; Ref, reference; VTE, venous thromboembolism.

### Rates of total body weight loss and associations with 12 months 25% total body weight loss

Overall, the mean (s.d.) 12-month TBWL for all procedures was 30.27 (11.58)%. The multivariate regression results for 25% TBWL are presented in Table 4. OAGB is associated with increased weight loss compared to SG and RYGB at the 12-month time point. Increased weight loss was noted in all phases of the institution caseload, but increased volume was associated with a higher likelihood of being associated with 25% total body weight loss at 12 months. Increasing age at surgery, increased ASA score, and T2D were associated with a reduced likelihood of achieving 25% TBWL at 12 months. No technical elements were independently associated with TBWL.

**Table 4:** Multivariate logistic regression of factors associated with achieving 25% total body weight loss at 12 months postoperatively for patients undergoing primary BMS in the NBSR.

	Adjusted OR (95% CI)	*P*
Procedure		
Sleeve gastrectomy	Ref	
OAGB (Cases 0–24)	2.04 (1.41–2.95)	<0.001
OAGB (Cases 25–49)	2.10 (1.37–3.22)	0.001
OAGB (Case 50+)	3.47 (2.75–4.39)	<0.001
RYGB	2.71 (2.54–2.90)	<0.001
AGB	0.16 (0.15–0.18)	<0.001
Other	0.42 (0.21–0.83)	0.013
Year of surgery		
2009–2012	Ref	
2013–2015	0.97 (0.91–1.04)	0.461
2016–2019	1.09 (1.01–1.18)	0.024
Funding type		
Self-pay	Ref	
NHS	0.90 (0.83–0.98)	0.013
Age at surgery (years)		
18–30	(Ref)	
31–40	0.85 (0.76–0.95)	0.006
41–50	0.72 (0.65–0.80)	<0.001
51–60	0.61 (0.54–0.68)	<0.001
60+	0.51 (0.44–0.58)	<0.001
Sex		
Male	0.98 (0.92–1.06)	0.652
Ethnicity		
Non-Caucasian	0.71 (0.65–0.77)	<0.001
Initial BMI (kg/m^2^)		
35–39.9	Ref	
40–49.9	1.49 (1.36–1.62)	<0.001
≥50	1.94 (1.76–2.14)	<0.001
Medical conditions		
Asthma	1.00 (0.93–1.07)	0.989
Cardiovascular disease	1.06 (0.93–1.20)	0.416
Depression	1.00 (1.00–1.00)	0.215
Dyslipidaemia	0.96 (0.89–1.03)	0.231
GERD	0.93 (0.87–0.99)	0.030
Hypertension	0.94 (0.88–1.01)	0.086
Liver disease	1.02 (0.92–1.15)	0.671
Musculoskeletal pain	1.00 (0.94–1.07)	0.892
OSA	0.96 (0.89–1.03)	0.241
VTE risk factors	0.87 (0.81–0.94)	<0.001
T2DM	0.69 (0.64–0.74)	<0.001
ASA		
1	Ref	
2	0.96 (0.87–1.07)	0.480
3	0.80 (0.71–0.90)	<0.001
4	0.72 (0.49–1.07)	0.102
Impaired functional status		
Cannot climb three flight of stairs or more	1.04 (0.97–1.11)	0.235

Impaired functional status defined as inability to climb 3 flights of stairs.

GERD, Gastroesophageal Reflux Disease; OR, odds ratio; OSA, obstructive sleep apnea; Ref, reference; VTE, venous thromboembolism.

### Factors associated with selection for OAGB and other procedures

When sleeve gastrectomy was used as the reference procedure, RYGB and OAGB were significantly more likely to be performed in patients with T2DM (Table 5), and gastric banding was significantly less likely to be performed. OAGB was more likely to be used than all other procedure types for patients with functional impairment and who were unwell, whether classified by ASA or OS-MRS score. Patients with GERD are more likely to be selected for RYGB. For both RYGB and OAGB, there was no significant effect of BMI after adjusting for other factors, whereas gastric banding was less likely to be used with a higher BMI. Male patients were more likely to undergo sleeve gastrectomy, older patients were less likely to receive RYGB when compared to SG and OAGB, and AGB was more likely to be used for older patients.

**Table 5:** Multinomial regression for of the associations with procedure type in NBSR patients undergoing primary surgery.

	OAGB		RYGB		AGB	
	OR (95% CI)	*P*	OR (95% CI)	*P*	OR (95% CI)	*P*
Age 60 years +	0.87 (0.75–1.01)	0.072	0.79 (0.73–0.85)	<0.001	1.23 (1.11–1.37)	<0.001
Male Sex	0.85 (0.77–0.95)	0.003	0.83 (0.79–0.87)	<0.001	0.64 (0.59–0.69)	<0.001
BMI 50+kg/m^2^	0.92 (0.84–1.01)	0.073	0.97 (0.93–1.01)	0.174	0.38 (0.35–0.41)	<0.001
GERD	0.84 (0.75–0.93)	0.001	1.82 (1.73–1.90)	<0.001	1.02 (0.95–1.10)	0.598
T2DM	1.40 (1.27–1.55)	<0.001	1.82 (1.74–1.91)	<0.001	0.71 (0.65–0.77)	<0.001
Functional impairment: cannot climb three flights stairs	1.19 (1.08–1.31)	<0.001	0.87 (0.84–0.91)	<0.001	0.65 (0.61–0.69)	<0.001
ASA 3+	1.30 (1.18–1.42)	<0.001	0.88 (0.84–0.92)	<0.001	0.48 (0.44–0.52)	<0.001
OS-MRS (High Risk)	1.56 (1.31–1.87)	<0.001	0.63 (0.57–0.69)	<0.001	0.88 (0.74–1.04)	0.135

Reference procedure: sleeve gastrectomy. Adjusted for ethnicity and year of surgery.

ASA, American Society of Anesthesiology score; GERD, Gastroesophageal reflux disease; OR, odds ratio; OS-MRS, Obesity Surgery Mortality Risk Score; T2D, type 2 diabetes.

### Impact of OAGB use on institutional bariatric practice

Thirty-four institutions were defined as OAGB performers and 138 institutions were defined as non-OAGB performers. A comparison of patient characteristics between these institutions is shown in Table 6. In the OAGB performer institutions, patients undergoing RYGB and SG prior to introducing OAGB had a higher proportion of male patients, those with higher BMI, and higher ASA, EOSS, and OS-MRS scores when compared to patients in non-OAGB-performing institutions. Additionally, SG patients were older among the OAGB performers. After the introduction of OAGB, the proportion of male patients, those with higher BMI, and those with elevated EOSS and OS-MRS scores were reduced for both RYGB and SG. There were fewer notable differences in patients with AGB between institution types.

**Table 6:** Impact of OAGB introduction in OAGB performing institutions on primary LSG, RYGB, and AGB patient preoperative characteristics compared with non-OAGB performing institutions in the NBSR.

	RYGB			SG			AGB			
	Pre-OAGB	Post-OAGB	Non-OAGB	Pre-OAGB	Post-OAGB	Non-OAGB	Pre-OAGB	Post-OAGB	Non-OAGB	*P*
Sex										
Male	3161 (23.3%)	1771 (20.1%)	7336 (20.3%)	1564 (27.7%)	1857 (23.3%)	7336 (20.3%)	465 (15.9%)	146 (14.9%)	7336 (20.3%)	0.025
Age (years)										
18–30	1353 (10.0%)	882 (10.0%)	4281 (11.9%)	659 (11.7%)	1059 (13.3%)	4281 (11.9%)	434 (14.9%)	171 (17.4%)	4281 (11.9%)	0.343
31–40	3020 (22.3%)	1954 (22.2%)	8298 (23.0%)	1244 (22.1%)	1900 (23.9%)	8298 (23.0%)	781 (26.8%)	219 (22.3%)	8298 (23.0%)	0.014
41–50	4845 (35.7%)	2838 (32.3%)	11735 (32.5%)	1848 (32.8%)	2350 (29.5%)	11735 (32.5%)	933 (32.0%)	295 (30.1%)	11 735 (32.5%)	<0.001
51–60	3444 (25.4%)	2367 (26.9%)	9030 (25.0%)	1385 (24.6%)	1982 (24.9%)	9030 (25.0%)	547 (18.7%)	217 (22.1%)	9030 (25.0%)	<0.001
61+	900 (6.6%)	757 (8.6%)	2754 (7.6%)	504 (8.9%)	666 (8.4%)	2754 (7.6%)	224 (7.7%)	78 (8.0%)	2754 (7.6%)	0.014
BMI (kg/m^2^)										
30.0–34.9	356 (3.0%)	481 (5.8%)	1674 (5.4%)	207 (4.0%)	393 (5.2%)	1674 (5.4%)	219 (8.9%)	155 (16.7%)	1674 (5.4%)	<0.001
35.0–39.9	1623 (13.4%)	1722 (20.7%)	5978 (19.2%)	810 (15.7%)	1509 (20.1%)	5978 (19.2%)	569 (23.1%)	258 (27.8%)	5978 (19.2%)	0.006
40.0–49.9	5968 (49.5%)	4095 (49.3%)	15539 (49.9%)	2333 (45.3%)	3464 (46.1%)	15539 (49.9%)	993 (40.3%)	357 (38.5%)	15 539 (49.9%)	0.018
50.0–59.9	2936 (24.3%)	1160 (14.0%)	6493 (20.8%)	1255 (24.4%)	1454 (19.3%)	6493 (20.8%)	264 (10.7%)	115 (12.4%)	6493 (20.8%)	<0.001
60.0 +	529 (4.4%)	137 (1.7%)	1308 (4.2%)	426 (8.3%)	436 (5.8%)	1308 (4.2%)	20 (0.8%)	9 (1.0%)	1308 (4.2%)	<0.001
ASA										
III	3981 (29.4%)	3195 (36.3%)	8597 (23.8%)	1759 (31.2%)	2964 (37.3%)	8597 (23.8%)	399 (13.7%)	159 (16.2%)	8597 (23.8%)	<0.001
IV	63 (0.5%)	54 (0.6%)	128 (0.4%)	39 (0.7%)	66 (0.8%)	128 (0.4%)	10 (0.3%)	5 (0.5%)	128 (0.4%)	<0.001
EOSS										
0	927 (8.1%)	469 (6.2%)	4334 (14.0%)	521 (10.4%)	701 (9.7%)	4334 (14.0%)	338 (16.4%)	137 (15.3%)	4334 (14.0%)	<0.001
1	807 (7.1%)	548 (7.2%)	2783 (9.0%)	409 (8.1%)	744 (10.3%)	2783 (9.0%)	331 (16.0%)	130 (14.5%)	2783 (9.0%)	0.007
2	8801 (77.1%)	6077 (80.0%)	22060 (71.2%)	3618 (71.9%)	5301 (73.1%)	22 060 (71.2%)	1318 (63.8%)	583 (65.2%)	22 060 (71.2%)	0.037
3	544 (4.8%)	345 (4.5%)	1189 (3.8%)	287 (5.7%)	286 (3.9%)	1189 (3.8%)	52 (2.5%)	29 (3.2%)	1189 (3.8%)	<0.001
4	333 (2.9%)	156 (2.1%)	626 (2.0%)	196 (3.9%)	224 (3.1%)	626 (2.0%)	26 (1.3%)	15 (1.7%)	626 (2.0%)	0.041
OS-MRS										
Low risk (0/1)	5323 (46.3%)	4478 (55.2%)	16735 (57.2%)	2250 (44.6%)	4079 (54.5%)	16 735 (57.2%)	1521 (63.5%)	605 (66.1%)	16 735 (57.2%)	0.048
Medium risk (2/3)	5443 (47.4%)	3349 (41.3%)	11 383 (38.9%)	2334 (46.3%)	2972 (39.7%)	11 383 (38.9%)	812 (33.9%)	283 (30.9%)	11 383 (38.9%)	0.003
High risk (4/5)	719 (6.3%)	288 (3.5%)	1130 (3.9%)	461 (9.1%)	432 (5.8%)	1130 (3.9%)	63 (2.6%)	27 (3.0%)	1130 (3.9%)	0.027

Column proportions were statistically compared by *χ* ^2^ analysis.

### Impact of OAGB and other procedure types on comorbidity changes

The overall rates and adjusted odds ratios for comorbidity changes after various bariatric procedures are shown in Table 7. OAGB had comparable comorbidity change data to sleeve gastrectomy for all reported comorbidities. RYGB showed improved comorbidity resolution, particularly for GERD, compared with all other procedures. AGB reduced the odds of T2D, hypertension, sleep apnea, and asthma remission. De novo GERD occurred in approximately a quarter of sleeve gastrectomy and OAGB procedures and in less than 20% of RYGB procedures.

**Table 7:** Rates and multivariate regression for procedure type associations with comorbidity changes in NBSR patients undergoing primary surgery.

	SG		OAGB		RYGB		AGB	
	Rate (n/n; (%))	OR (95% CI)	Rate (n/n; (%))	OR (95% CI)	Rate (n/n; (%))	OR (95% CI)	Rate (n/n; (%))	OR (95% CI)
T2D remission	1458/2305 (63.3%)	REF	209/332 (63.0%)	1.04 (0.81–1.34)	3958/5735 (67.0%)	1.22 (1.10–1.36)	278/651 (42.7%)	0.38 (0.31–0.45)
Hypertension remission	19 78/3926 (50.4%)	REF	262/524 (50.0%)	1.05 (0.87–1.27)	4588/7749 (59.2%)	1.33 (1.22–1.44)	456/1244 (36.7%)	0.49 (0.42–0.56)
OSA remission	1631/2711 (60.2%)	REF	204/330 (61.8%)	1.27 (0.92–1.75)	3241/4745 (68.3%)	1.31 (1.15–1.49)	247/534 (46.3%)	0.46 (0.36–0.58)
Asthma remission	964/2085 (46.2%)	REF	158/319 (49.5%)	1.11 (0.87–1.41)	2131/4096 (52.0%)	1.23 (1.10–1.37)	285/736 (38.7%)	0.70 (0.58–0.84)
GERD remission	1052/2108 (49.9%)	REF	112/218 (51.4%)	1.09 (0.82–1.45)	3678/5407 (68.0%)	2.03 (1.82–2.26)	459/871 (52.7%)	1.19 (1.00–1.42)
De Novo GERD	2076/8024 (25.9%)	REF	240/878 (27.3%)	0.98 (0.83–1.15)	2287/13181 (17.4%)	0.55 (0.51–0.59)	295/3631 (81%)	0.29 (0.26–0.34)

Reference procedure: Sleeve gastrectomy. Adjusted for age, sex, preoperative BMI, ethnicity, ASA score, OS-MRS score, and type of healthcare funding (NHS/private).

ASA, American Society of Anesthesiology score; GERD, gastroesophageal reflux disease; OR, odds ratio; OS-MRS, Obesity Surgery Mortality Risk Score; T2D, type 2 diabetes.

## Discussion

This study uniquely reported the introduction of a newer bariatric procedure, one-anastomosis gastric bypass, into the national bariatric surgery practice in the United Kingdom. OAGB has been shown to reduce the risk of complications, including severe complications, even during the initial phases of clinical use. This is particularly notable, as the OAGB population had higher rates of functional impairment and disease complexity, as measured by the obesity surgery-metabolic risk score. The rates of weight loss at 12 months were higher than those reported for either RYGB or SG and did not appear to be affected by the stage of institutional experience with the technique. Patients with greater functional impairment and higher levels of obesity-related unwellness appear to be selected for OAGB. This reflects the change in practice in institutions that use OAGB, where the proportion of this patient cohort undergoing alternative bariatric surgery procedures is reduced.

In this study, OAGB was shown to be independently associated with a reduced risk of perioperative complications compared to other primary bariatric procedures in the NBSR dataset. This finding is in agreement with the results of other studies. The GENEVA international cohort of 6770 patients from 185 centers undergoing bariatric surgery during the COVID pandemic reported that RYGB had higher rates of perioperative complications compared to SG and OAGB^28^. Propensity-matching scoring analysis from the Metabolic and Bariatric Surgery Accreditation and Quality Improvement Program (MBSAQIP) showed that OAGB had a lower complication rate than RYGB, but no statistical difference compared to SG^29^. However, in that study, only 341 cases were included, which reflects the status of OAGB as a non-ASMBS-accredited procedure at that time. Data from the YOMEGA randomised trial^30^ showed that the severe complication rate requiring reoperation was half that for patients undergoing RYGB, which agrees with this study’s multivariate analysis for severe complications. The reasons for reduced morbidity with OAGB are likely to be related to the comparatively reduced technical challenge of the performance of the procedure. Data from the MBSAQIP registry and the YOMEGA randomized trial (for OAGB versus RYGB) reported shorter operating times with OAGB than with RYGB and SG. The present study demonstrates that this may have impacted patient and surgical decision-making for procedure selection. Patient characteristics associated with fragility and increasing surgical technical challenges, including higher age, BMI greater than 60 kg/m^2^, and elevated ASA, OS-MRS, and EOSS scores, were reduced in RYGB and SG patients in institutions that introduced OAGB. This study extends the finding of reduced perioperative morbidity with larger numbers of OAGB patients undergoing primary surgery in routine clinical practice, including the introduction and learning phases in individual institutions.

In the comparison of 12-month weight loss, OAGB was shown to be independently associated with higher total body weight loss when compared to other NBSR procedures. Similarly, a recent network meta-analysis incorporating 20 RCTs of 1803 patients indicated higher excess weight loss in patients undergoing OAGB than in those undergoing RYGB and SG^14^. Data from the International Federation for Surgery for Obesity Global Registry indicated a slightly higher percentage of excess weight loss at 1-year follow-up for OAGB than for RYGB, SG, and AGB^31^. The current study also demonstrated improved 12-month weight loss for RYGB compared to SG. This agrees with recent work from combined Swedish, Norwegian, and Dutch quality registries^32^. The present analysis also shows that increased institutional experience with OAGB is associated with improvements in this domain.

The safety of OAGB during the introductory phase, particularly in comparison with other bariatric procedures, is a unique finding in the literature. There is widespread acknowledgement that the unrestricted introduction of novel surgical procedures can place patient safety at risk, as shown with laparoscopic cholecystectomy^33^ and jejuno-ileal bypass for obesity^34^ in the 20th century. There is a paucity of studies examining the introduction of new surgical techniques into the generality of bariatric practice. A study by Burns *et al*.^35^ using administrative data demonstrated that the early use of sleeve gastrectomy was not associated with a higher 28-day readmission or 30-day mortality rate; however, reoperation was not included in that study, and there are acknowledged challenges with identifying procedure type, particularly new techniques, from administrative coding. Data from the Scandinavian Obesity Surgery Registry (SOREG) has shown that institutions with a learning curve for laparoscopic RYGB were associated with an increased risk of serious complications^36^. The learning curve in this study was defined as the total institutional experience of up to 400 operations. The SOREG study noted that in the first 100 cases, the rates of complications were not statistically different from established practice (500 cases onwards) and postulated that this could be secondary to mentoring effects and the selection of less technically challenging individuals. In this study, the median institutional experience with RYGB was around 350 operations prior to starting OAGB, which may additionally explain the absence of a significant perioperative morbidity signal associated with the introduction of a new surgical procedure, despite the patients undergoing OAGB being older and with more severe metabolic disease.

The study has several strengths, including case ascertainment of over 95% compared to NHS administrative data, and the data are from the UK NBSR, a comprehensive data registry with mandatory annual reporting^37^. This study has some limitations that merit consideration. Primarily, there is no coding in NBSR to indicate whether mentoring or training prior to the introduction of a new procedure has taken place; therefore, this study cannot comment on this important context of introducing a new surgical technique. Second, there was no specific entry option for OAGB in the NBSR until 2016, when an updated database version was released, with OAGB operations entered prior to this date identified from contemporaneous free text entry by the operating surgeons. As a result, fewer specific technical operative elements are available for analysis prior to this date. Third, it is possible that patients presented to alternative institutions with complications; therefore, these may not have been coded in NBSR. However, this would likely affect all procedures similarly, and the specialist nature of bariatric surgery in the UK means that people who have undergone bariatric surgery are most likely to present to their operating centers in the first instance. Fourthly, our clinician-reported dataset only captures monthly follow-up information for around 40% of the cohort, which is consistent with other large bariatric registries, such as the MBSAQIP, but is less than some other registries, such as those present in Scandinavia, which have linkage to individual patient identifiers. Therefore, the 12-month findings from this study should be confirmed in other larger registries where OAGB has been introduced. Finally, the use of bariatric surgery in UK practice, particularly within the NHS, is often late in the stage of metabolic disease, often with multimorbidity^23,38^. This may not be reflective of global obesity surgery practice; therefore, OAGB may not impact national bariatric practice in other healthcare systems in the same manner. However, the registry data from both Israel^19^ and Italy^39^ indicate that the increasing use of OAGB points to a degree of generalizability.

This study has shown that OAGB has been incorporated into national bariatric practice in the United Kingdom without significant morbidity. OAGB use is associated with reduced perioperative morbidity and is potentially associated with greater weight loss at 12 months, despite the fact that patients with older age and greater obesity-associated disease were selected for the procedure. This has impacted the complexity of patients who receive alternative procedures in hospitals that use OAGB. Careful ongoing assessment of use of a new procedure has been shown to be possible within the context of a national registry.

## Ethics approval

Not applicable.

## Consent

Not applicable.

## Source of funding

Not applicable.

## Author contribution

A.C.C., A.A., P.S., and K.M.: conceptualized the study; A.C.C. and A.A.: conducted the statistical analyses; A.C.C., A.A., J.B., O.A.K., P.S., and K.M.: drafted the manuscript. All authors critically revised the manuscript and agreed to the final version.

## Conflicts of interest disclosure

The authors declares no conflicts of interest.

## Research registration unique identifying number (UIN)

Registered retrospectively in clinicaltrails.gov : NCT06167005.

## Guarantor

Andrew Currie.

## Data availability statement

There are restrictions on the availability of data for this study because the initial patient consent forms only allow the sharing of data for research purposes. Researchers wishing to access an anonymized dataset containing individual participant data can apply to the National Bariatric Surgery Registry at info@bomss.org where the data are stored in a repository.

## Provenance and peer review

Not commissioned, externally peer-reviewed.

## Supplementary Material

SUPPLEMENTARY MATERIAL
