# Supplementary material for: National introduction of one-anastomosis gastric bypass in the UK National Bariatric Surgery Registry: a cohort study
Source: Int J Surg. 2024 Sep 23;110(12):7404–13. doi: 10.1097/JS9.0000000000002005 (PMC11634166; doi:10.1097/JS9.0000000000002005)
Supplement: SUPPLEMENTARY MATERIAL [file js9-110-7404-s002.docx]

**Supplementary file 1. EOSS score generation from the NBSR**

| **EOSS stage** | **Comorbidity** |
| --- | --- |
| **4**  *One or more of the following characteristics* | Requires a wheelchair or is housebound |
|  | Has venous oedema with ulceration |
|  | Has had a vena cava filter |
|  | Obesity/hypoventilation syndrome |
|  | Liver cirrhosis |
|  | |
| **3**  *If no criteria for EOSS stage 4 are met, one or more of the following qualifies as EOSS 3* | Diagnosed with atherosclerosis |
|  | Sleep apnoea with complications |
|  | Asthma requiring treatment with nebulisers or oral steroids or requiring hospital admission in the last year |
|  | Known arthritis / back or leg pain from arthritis requiring opiates |
|  | Non-alcoholic steatohepatosis proven on liver biopsy |
|  | Infertility (female patients only) |
|  | |
| **2**  *If no criteria for EOSS stages 3 and 4 are met, one or more of the following qualifies as EOSS 2* | Oral hypoglycemic or insulin therapy for type 2 diabetes |
|  | Hypertension on treatment |
|  | Dyslipidemia |
|  | Diagnosis of sleep apnoea or on CPAP/BiPAP |
|  | Asthma treated with regular inhalers |
|  | Can climb half a flight of stairs without resting |
|  | Back or leg pain from arthritis requiring regular medication with non-opiates |
|  | Daily medication with H2RA/PPI or previous anti-reflux disease |
|  | A prior operation for reflux disease |
|  | Known non-alcoholic fatty liver disease proven on biopsy or hepatology opinion |
|  | Depression on medication |
|  | On medication for polycystic ovary syndrome (female patients only) |
|  | |
| **1**  *If no criteria for EOSS stages 2, 3 and 4 are met, one or more of the following qualifies as EOSS 1* | Impaired glycaemia or impaired glucose tolerance |
|  | Intermittent symptoms of back or leg pain from arthritis not treated with medication |
|  | Can climb 1 flight of stairs without resting |
|  | Suspected non-alcoholic fatty liver disease (abnormal liver function tests or abnormal ultrasound scan) |
|  | Diagnosis of polycystic ovary syndrome that is not treated with medication (females only) |
|  | Intermittent medications or intermittent symptoms of reflux disease |
|  | |
| **0**  *No criteria for EOSS staged 1-4 are met* |  |
