# Supplementary material for: National introduction of one-anastomosis gastric bypass in the UK National Bariatric Surgery Registry: a cohort study
Source: Int J Surg. 2024 Sep 23;110(12):7404–13. doi: 10.1097/JS9.0000000000002005 (PMC11634166; doi:10.1097/JS9.0000000000002005)
Supplement: SUPPLEMENTARY MATERIAL [file js9-110-7404-s003.docx]

Appendix 1. Method of identification of OAGB cases from NBSR version 1 prior to 2016.

The OAGB operations were derived from the free text field DetailsOfOtherOperations version 1 of the National Bariatric Surgery Registry.

The definition of OAGB /MGB was:

The value of the free text field matched any of following:

"*fied scopin*",

"*dge*loop*",

"*MBG*",

"*pic*single*anas*",

"*sagb*",

"*omega loop*",

"*loop gastr*",

"*OAGB*",

"*mini*",

"*ss sing**sis*",

"*sing*an*sis*g*",

"*sing*an*sis*b*",

"*MGB*",

"*one*an*si*",

"*single loop ana*sis*",

"*single an*sis loop*",

"*single*loop*bypass*",

"*min*gas*",

"single a*astom*",

"loop*bypass"

  The value of the free text field did not match any of the following:

"*reversal*",

"*minimiz*",

"*trimming*"
